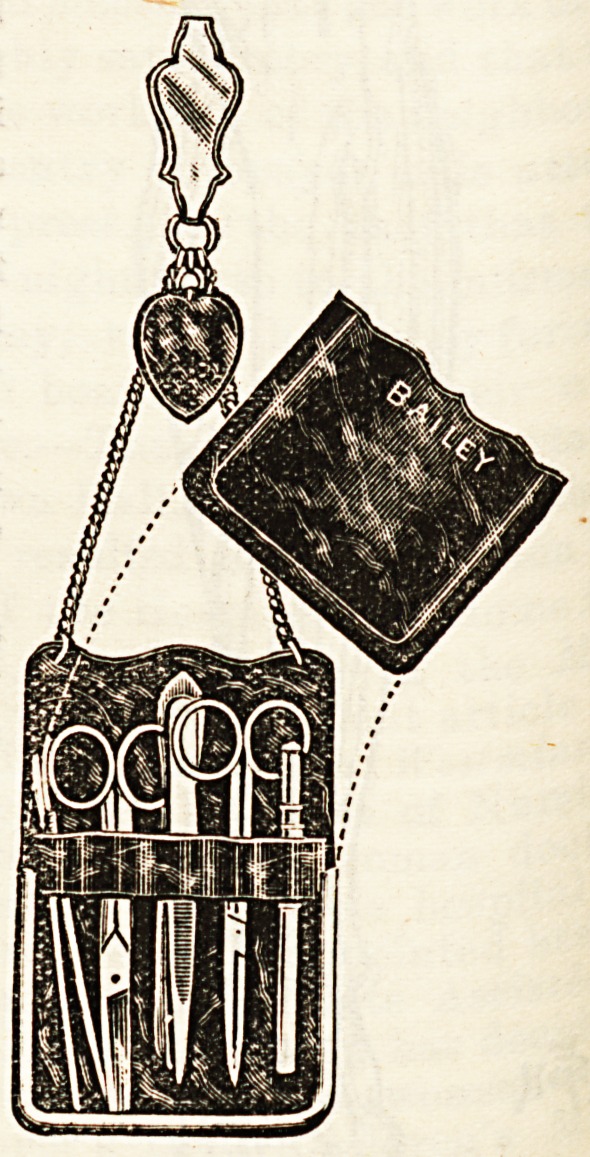# The Hospital Nursing Supplement

**Published:** 1895-05-25

**Authors:** 


"The Hospital" Nursing Mirror.

Being the Extra Nursing Supplement of "The Hospital" Newspaper.

[Contributions for this Supplement should be addressed to the Editor, The Hospital, 428, Strand, London, W.C., and should have the word "Nursing" plainly written in left-hand top corner of the envelope.]

### News from the Nursing World.

### THE PRINCE OF WALES.

The Warwick Workhouse was honoured by the inspection of the Prince of Wales last Monday. After going round the wards His Royal Highness wrote in the visitors' book, "I have visited this union with the greatest interest and have found everything very clean, the inmates being well-cared for, and everything in excellent order." Mr. Stanton, Chairman of the Board, and the Countess of Warwick, who is herself a most energetic Guardian, accompanied the Prince.

### "WORK IS AS IT IS DONE."

The students of the London School of Medicine for Women and the Royal Free Hospital have made a new departure this month by bringing out a magazine of their own. The rising generation of students has secured the co-operation and encouragement of qualified women doctors in their literary venture, and the motto placed on the cover of the first number, "Work is as it is done," is one full of promise. Amongst hospital news a pleasant description is given of a tea and social evening at which the ward sisters were entertained by the women students.

### THE MIDWIVES BILL.

At the meeting of the Women's Liberal Federation last week the registration of midwives was exhaustively discussed. The principle of compulsory registration appeared to be generally approved of, although many speakers criticised the present Bill very freely. One of the speakers acknowledged the great help given by Sir Walter Foster, who spoke eloquently in favour of the first Midwives Bill at its introduction into the House of Commons. Hopes were expressed that he would see his way to supporting the present Bill, which has been drawn up by the Midwives' Board, consisting of twelve doctors. At the close of the meeting the following resolution was passed by a large majority: "That as the absence of any State regulation of midwives leads to the practice of midwifery by unqualified and incompetent women, and is therefore fraught with extreme danger to the lives of mothers and their infants, this Council heartily supports the principle of the Bill for the Registration of Midwives, which has just passed its second reading in the House of Lords, and urges Her Majesty's Government to give its support to the same."

### WOMEN INSPECTORS.

The Islington Vestry have appointed Miss Jessie Gray as their Inspector of Women's Workshops, at a salary of £150 per annum. In addition to training as a nurse at Addenbrooke's Hospital, Miss Gray has passed the examination of the Sanitary Institute. The Kensington Vestry has recently conferred upon its senior woman inspector the full powers of a sanitary inspector. This step was advised by a committee some time ago, and the work of the two ladies whom the Kensington Vestry engaged as inspectors a year and a half since appears, from Dr. Dudfield's report, to have proved eminently satisfactory.

### OPINIONS DIFFER!

"I've been doin' for 'im for nigh upon thirty year, and I'm just about sick of 'im!" replied the married woman, who had drifted from the infirmary into the workhouse. The airy cubicles designed for the occupation of married couples had no attraction for her, and she disappeared contentedly into the women's ward as a haven where she would be relieved of the burden of "doin' for 'im!" The incident startled a lady visitor accustomed to discussing the "cruel separation" of old couples in workhouses, but another disillusion awaited her when she sympathised with an aged pauper encountered outside the workhouse, on the hardship of "the pauper's garb." The woman turned on her sharply, and in language unsuitable for reproduction declared that any amelioration of the uniform would be extremely unwelcome, as it had a distinct financial value in the eyes of those who, like herself, devoted every hour she was "allowed out" to the solicitation of alms for the "drop o' gin or 'bacca," which would be probably withheld if her costume appealed less strongly to the sympathies of the kindly passers-by.

### CARD TRICKS UP TO DATE.

The plain visiting card often left with employers or handed to doctors by such trained nurses as have already proved to both the efficiency of their services appears to have given rise to imitation of a most undesirable kind. These pieces of innocent-looking pasteboard may bear only the name and address of "Nurse" So-and-So, leaving the task of investigation of the applicant's credentials optional. Others, however, are decorated with various symbolic initials which generally carry more weight with the unprofessional recipient of the card than the title "trained nurse" placed in modest brackets in a corner. Unluckily for the confiding employer, it not unfrequently happens that the person thus described is eventually discovered to found her pretensions on a few weeks' training in monthly work or an equally limited experience in some small institution where her services were not long considered acceptable. Besides this bogus "trained nurse" there is another type, the nurse who seeks to add to her own value by the addition to her address of the name of some recognised institution or association. Inquiry will sometimes show that her connection with such is either purely imaginary or has long been severed. The only protection which the public possesses lies in the power to appeal to any association for confirmation of asserted facts, at the expense of time and trouble when delay is inconvenient. Much will be done to lessen the dangers of this "card trick" when the *Official Directory* is published, by means of which the public will be able to ascertain at once, from official sources, what sort of training each nurse has undergone.

### LONG HOURS.

The long hours of asylum attendants recently discussed in the lay press appear to demand prompt remedies. The daily "two hours off duty" which the best hospital schools give to their nurses should be granted to attendants. So long as some general hospitals most injudiciously keep their nurses on duty in sick wards for the whole of two or three consecutive days, the asylum authorities will find excuses for their own action. Attendants often get a great deal of fresh air and exercise in the course of their duty, but even this does not compensate them for having to spend every moment of a working day in close companionship with patients who are mentally afflicted.

### AMBULANCE WORK AT SHREWSBURY.

The formation of a Shrewsbury Branch of the St. John Ambulance Brigade has been resolved upon, with the object of enabling those who have obtained a "First Aid" certificate to keep up and utilise their knowledge. Mr. F. E. Adams has been elected superintendent, and an influential committee has been formed. Some combination with district nursing was mentioned at the meeting as desirable, and there is no doubt that the engagement of a thoroughly trained woman would be of practical utility. Mr. Adams appears to possess wholesome views with regard to the assumption of "uniform" by amateurs, speaking of it as "unnecessary," a view which most Hospital readers will cordially endorse.

### PLEADING FOR THE CHILDREN.

In the Section of State Medicine of the Royal Academy of Medicine in Ireland an interesting discussion took place last month respecting the early training of children. Several physicians spoke of the effect on the after-life of individuals of the lessons received in childhood, Dr. Grimshaw remarking that babies could be educated from their cradle. Dr. Bernard spoke approvingly of the work of the Queen's Nurses, who had "cleared the way for ladies to visit the poor people, and teach them cooking, thrift, and cleanliness." The opinions of those so well qualified to judge of the necessity for early moral and physical training should carry weight even with boards who hold primitive views respecting their responsibilities as Guardians of the Poor. The "early impressions" in workhouse nurseries are sometimes derived exclusively from imbeciles and other equally unsuitable attendants. In spite of the increased value set on the lives of children, of which Dr. J. W. Moore spoke at this Irish meeting, the care of them in sickness and health does not always command the consideration of intelligent women, being too frequently left to the grudging care of most ignorant persons.

### ABERDEEN DISTRICT NURSES.

The removal of these district nurses to their new home on Castle Hill will take place next week, but certain needed alterations will not be completed before July. The Aberdeen District Nursing Association is affiliated with the Scottish Branch of the Queen's Jubilee Institute, and the General Inspector reports "that she has seen no work better done than in Aberdeen, nor more thoughtful care and kindness bestowed on the patients." The work is increasing, and the appreciation which it receives from the poor is of a most cordial character. Miss Armstrong may be congratulated on the success of the movement which her own ability and devotion have been chiefly instrumental in forwarding.

### ASYLUM ATTENDANTS.

Six male and seven female attendants at Carmarthen Asylum passed the recent examination of the Medico-Psychological Association, Dr. Pringle, medical superintendent of Glamorgan County Asylum, being the examiner, and Dr. Goodall, of the Carmarthen Asylum, assessor on the occasion. To the latter much credit is due for the excellent instruction which enabled the staff to distinguish themselves most satisfactorily at the recent examination.

### TRAINING ESSENTIAL AT LUCKNOW.

The days seem happily to be passing away when the alliance of "some experience" of sick nursing with "the missionary spirit" was deemed sufficient for hospital work abroad. In many institutions there are still, doubtless, women holding positions of authority, and subjected to a minimum of supervision, whose theoretical and practical knowledge of the nursing art is conspicuously absent. But fuller preparation is demanded of the recruits, and in the report of the Kinnaird Memorial Hospital, at Lucknow, in this month's issue of "The Zenana," Miss Haskew writes: "Our great desire is to make our wards a thoroughly good nursing school." For the attainment of this end a thoroughly trained English nurse is now required. The present matron is said to be "doing her best, but she has not had a training, and no amount of willingness can make up for lack of drill." The patients are each allowed to have the company of a friend or relation during the whole time spent in hospital, and there are other characteristics of hospital administration in the Indian Empire which demand tact on the part of the nurse as well as intelligence and skill.

### SHORT ITEMS.

The West Hartlepool and District Nursing Association reported at the annual meeting that the work of its three nurses was thoroughly satisfactory, and that the hearty co-operation of the workmen of the neighbourhood continued.—A Coventry ratepayer calls attention, through the local press, to the fact that the guardians who refused a night nurse to the infirmary on the ground of economy, voted the money for enlarging and refitting the board-room for their own comfort and convenience.—The May Day entertainment at Kensington Town Hall, which was organised by Miss Jacombe-Hood, resulted in the addition of about £63 to the funds of the Hospital and Home for Incurable Children, at 2, Maida Vale.—In the May number of *The Nurses' Journal*, an excellent article by Miss de Pledge is reprinted from the *Westminster Review*, on "The History and Progress of Nursing in Poor Law Infirmaries."—The first woman doctor to be placed on the staff of a Brussels hospital is Mademoiselle M. Derchird, recently appointed assistant physician at the Hôpital des Enfants Assistées.—In Russia the Education Department has decided to throw open all medical schools to women.—In connection with Mr. Boyer's Drift Children's Mission at Bow a seaside home has been opened at Margate in Cecil Square, for which contributions in money and furniture are required.

### Elementary Anatomy and Surgery for Nurses.

By W. McAdam Eccles, M.B., M.S., F.R.C.S., Lecturer to Nurses, West London Hospital, &c.

### XVIII.—THE SYSTEMIC CIRCULATION (*continued*).

### The Arteries of the Upper Extremity.

Coming from the innominate artery with the right common carotid is the right subclavian artery, while that of the left

Fig. 28.—The arteries of the upper extremity.

side springs directly from the aorta. Both subclavians arch over the first ribs, and then pass into the axillæ, or armpits, where they are termed the axillary arteries. In their course they can be felt pulsating above the clavicle, and can be easily compressed against the first rib. As already noted, the subclavian artery in the first part of its course gives off a very important branch, the vertebral. The axillary artery traverses the axilla, being deeply placed for the most part; at the lower border of that space it becomes the brachial artery. The course of this artery in the arm will be indicated by a line drawn from a point at the junction of the anterior third with the posterior two-thirds of the interval between the two folds of the axilla, to a spot half-way between the two condyles of the humerus, and one inch below the bend of the elbow, where the vessel bifurcates. (See Fig. 28.) Along its whole length it is comparatively superficial, and can be most readily compressed against the humerus. The two vessels into which it divides are the radial and ulnar. The radial artery runs down on the outer side of the forearm in an almost straight line from its point of origin to the spot where it can be felt pulsating at the wrist, this being called the radial pulse. Afterwards it turns outwards on the back of the hand to presently sink between the first and second metacarpal bones into the palm of the hand. The ulnar artery takes at first a curved course until it reaches the inner side of the forearm at the junction of the upper with the lower two-thirds, whence it descends vertically to the wrist, and then passes into the palm of the hand. The ulnar in its upper part gives off an artery to nourish the back of the forearm. Two arches are formed in the palm itself, one the superficial, about half-way down the palm, the other the deep, somewhat higher than this.

Fig. 29.—The arteries of the lower extremity.

### The Arteries of the Lower Extremity.

The external iliac passes into the upper part of the thigh at a spot which is half-way between the anterior superior spine of the ilium (Lect. IV.) and the symphysis pubis, where it may be compressed if necessary against the pubic bone. The artery in the thigh is called the femoral (see Fig. 29), and its course can be marked out when the limb is rotated outwards and slightly flexed by a line from the spot just indicated to a prominence which can be felt on the upper part of the inner condyle of the femur; the upper two-thirds of this line represents the femoral, and the lower third part of the popliteal artery, the continuation of the femoral. This vessel lies behind the knee joint, and about two inches below this joint divides into two, the anterior and posterior tibial arteries. The anterior tibial artery passes through between the tibia and fibula to the front of the leg on the outer side. (See Fig. 29.) It is at first deeply placed, but towards the lower part of the leg becomes much more superficial, and can be felt beating on the anterior surface of the ankle half-way between the two malleoli. It is continued on to the foot as the dorsal artery, which soon, however, sinks into the sole, passing between the first and second metatarsal bones. The posterior tibial continues apparently the course of the popliteal lying at the back of the leg on the inner side. It is also deeply situated at the upper part, where it gives off a large branch, the peroneal, which runs down on the outer side of the posterior surface of the leg close to the fibula. Near the inner ankle the posterior tibial can be easily felt pulsating, and soon divides into the external and internal plantar arteries which supply respectively the outer and inner side of the sole of the foot.

Most of the arteries which have been spoken of are accompanied by veins, but in addition to these deep veins there are a large number which run quite superficially, and owing to their great liability to become varicose some of these must be mentioned. In the upper extremity, if the arm be firmly seized above the elbow, several veins in front of that joint will stand out somewhat in the shape of the letter M. In some people the external jugular vein may be seen at the side of the neck, but the internal jugular is deeply placed alongside of the carotid artery. In the lower extremity the internal saphenous vein is frequently the seat of varicosity. This vein commences as an arch on the dorsum of the foot, and passing to the inner side ascends in front of the internal malleolus up the inner side of the leg, behind the inner condyle of the femur, and then along the inner side of the thigh to just below Poupart's ligament, a fibrous band which can be felt stretching between the anterior spine of the ilium and the spine of the os pubis.

### Queen Victoria's Jubilee Institute.

### ITS WORK AND WANTS.

SINCE the Queen determined to devote £70,000 of the offering of the women of England on the occasion of Her Majesty's Jubilee to secure a supply of skilled nurses in the homes of the poor, great progress has been made. Some impatience was manifested at the outset, because no immediate result was discernible. The scope of the trust covered the establishment of a system not merely for nursing the sick, but for introducing cleanliness, order, and sanitary conditions into the homes of the poor. It was not an easy thing to begin to interest rural populations in such a scheme, and it was even more difficult to introduce it in the towns. By persistent effort and devoted skill and attention, Mr. W. Rathbone, with the aid of the Council, has secured a remarkable success for the Queen's Institute, which must be very gratifying to Her Majesty.

One hundred and twenty-eight new associations have been established in connection and co-operation with the Institute, and each one of these is duly inspected, and the high standard of qualification is maintained. Altogether, the Institute has made grants to the extent of £3,638 to the new associations which now have an annual expenditure of about £28,000, and which nurse some 40,0C0 cases. Nor is this all, for ninety-six other associations have become affiliated, which are also inspected, and receive grants and expend £20,000 a year and nurse another 40,000 cases. It is a considerable financial feat on the part of Mr. Rathbone to have so arranged that by supplementing local efforts to the extent of some £5,000 only, nearly 80,000 cases have been nursed in the homes of the poor at an expenditure of nearly £50,000 per annum, all of which money is now given by the well-to-do neighbours of the poor in the localities where they reside. Here surely is good work of the highest kind quietly but most efficiently done.

The results are greater than the most sanguine could have anticipated to be possible. The management and organisation of the Institute are worthy of the latter part of the nineteenth century, and we congratulate all who have had any part in the work upon so magnificent an achievement. Much has been done, but even more remains to be accomplished. The present income of the Institute is too small to enable the Council to extend the work to every part of the United Kingdom. They require, and should at once receive, an additional income of £3,000 a year to enable them to start and encourage new associations, and to supply trained nurses. We hear a good deal in favour of Imperial Federation, and of the ties which bind all parts of the British Empire in a close embrace of partnership, good fellowship, and prosperity. We have also heard that there are gentlemen who have recently made enormous fortunes in connection with South Africa, who desire to find some great object of the widest social importance, to which they can give of their abundance a substantial sum. We commend to their attention the Queen Victoria's Jubilee Institute, St. Katherine's, Regent's Park, N.W., to which the Queen has herself given £70,000, and which is now in urgent need of £30,000 more, to make the whole organisation adequate to the needs of the poor who have to be nursed in their own homes in all parts of the United Kingdom.

### Appointments.

BRITISH HOSPITAL, MONTEVIDEO, SOUTH AMERICA.—Miss Charlotte E. Bright has received the appointment of Matron at this hospital. She was trained at the General Hospital, Wolverhampton; was afterwards ward sister at Prince Alfred Hospital, Sydney; lady superintendent of the Children's Hospital, Brisbane; matron of the Croydon District Hospital, South Queensland; and recently sister at the Strangers' Hospital, Rio de Janeiro. Miss Bright is to be congratulated on her new appointment, to which she takes the good wishes of all her friends.

### Care of the Sick in Alexandria and Cairo.

### II.—THE MILITARY HOSPITAL.

THIS hospital lies under the shadow and protection of Pharos, the first lighthouse ever built, which has been kept alight during the last twenty-eight years by an old Frenchman who lives in rooms adjoining with his wife and daughter. The hospital is a low one-storied building placed round a garden, and it has a quaint style of its own, although only raised to the dignity of a hospital some eight years ago; previously it was a stable for mules. The Netley sisters make their quarters dainty and home-like with their own little possessions, and from a verandah outside their rooms they get a beautiful view of the harbour. The floor was formerly covered with matting, but this attracted ants in such multitudes in spite of the legs of the table standing in water, that the only remedy was to banish the matting.

The side of the square opposite the sisters' quarters is occupied by wards, one labelled "Itch Ward," another "Lunatic," the first being used for any disease requiring isolation. The infectious cases are nursed by orderlies, who wear blue linen clothes, and are not allowed to communicate with any one while on duty. Navy sailors as well as soldiers are treated here by the army doctors.

The wards are long and narrow, with blue and red walls. They contain pictures, big screens, and stoves, while outside a covered verandah faces the garden, and as doors and windows are open, the whole place looks very cheerful. The nurses take their constitutionals on the ramparts, which are fine breezy places, commanding views of the sea.

There are still to be seen the damages wrought by the bombardment, at which the old French lighthouse keeper was present. We were much struck by the youthful appearance of the soldiers, many of them seeming mere boys as they looked up from their beds. Both doctors and nurses gave us the impression that the sick were well and skilfully cared for. The sisters are allowed a month's holiday each year, but generally save it up to three months, and then go home to England. Interesting, suitable books for the soldiers in hospital would, we fancy, be very acceptable.

### Novelties for Nurses.

(MESSRS. BAILEY AND SONS, OXFORD STREET, W.)

MESSRS. BAILEY AND SONS are one of the most enterprising firms in catering for the wants of nurses. All their new inventions and improvements are based upon practical knowledge of their wants. Anticipating that in the crusade against microbes the ordinary leather wallet or chatelaine might be regarded by some with suspicion, Messrs. Bailey have preserved the life of the wallet even in such circumstances by the manufacture of an article to meet the objections of the most scrupulous. The wallet is made of a horny substance in place of the leather. Thus lightness, durability, and cleanliness are secured. As shown in the illustration, the front of the receptacle slips off, and on removing the instruments the whole case can be rendered completely antiseptic by washing. The design before us is manufactured to represent tortoiseshell, but if nurses prefer a less conspicuous article they can obtain a neat wallet made to imitate leather. The cases have an excellent appearance, and will form a suitable and welcome present for those entering upon a nurse's career.

### Everybody's Opinion.

[Correspondence on all subjects is invited, but we cannot in any way be responsible for the opinions expressed by our correspondents. No communications can be entertained if the name and address of the correspondent is not given, or unless one side of the paper only be written on.]

### TRAINED NURSES AND ASYLUM ATTENDANTS.

Mr. H. B. Bardwell, member of the Visiting Committee, Devizes Asylum, writes: The objection I hear raised to the employment of trained nurses in lunatic asylums is that they and the attendants might not work well together; that jealousy and friction would be created. Can anyone give his experience on this? There is another question. Is the hospital accommodation in asylums for the patients and the attendants up to date? And are attendants for the most part nursed in their own little rooms? Are patients put into wards of limited space, that a fourth-rate country hospital would scorn and which would not be tolerated in a country workhouse? Is not this too much overlooked by the Lunacy Commissioners, and perhaps by the Visiting Committees? Your valuable paper, together with the advocacy of the profession, may alter some of this.

### A WARNING TO NURSES.

"Nurse W." writes: The Hospital of May 18th contained "a warning" from a nurse; and may I say that I think that nurse was wrong in not following the man in question? His wife might have been dangerously ill, and the fact of his betraying no distress at a refusal counts for nothing. Men are not wont to "wear their hearts on their sleeves." I have been a nurse for just over twenty years, and it has not been unusual for me to be stopped in the street. In some instances for very trivial causes, but on four different occasions my services were claimed in serious emergencies. On one occasion a little child's clothes had caught fire; on another a young man had fallen from a great height, and was terribly injured; a third time it was a poor woman taken in labour prematurely, who had been found by her husband with her dead babe when he went home to his dinner; the fourth time a lady was taken ill at a railway station, and her relations have not forgotten the little service I rendered on that day. A nurse, like a Religious Sister, should have no thought of self, and no fear of harm. She could quickly withdraw on suspecting any wrong were intended. I would always advise a nurse to respond to an appeal, and to mistrust no one without cause. I say further, in God's Name and in His work—trust all rather than none!

### CANTERBURY GUARDIANS AND RATEPAYERS.

"A Hospital Reader" writes: The Canterbury Board of Guardians have not improved their staff of nurses; instead of a trained nurse they have appointed a girl twenty-two years of age as head, with no training either in midwifery or hospital nursing; yet some young guardians thinking her wonderful, of course she got the appointment.

[We wrote to the clerk to the guardians on receiving this letter, asking whether we were correctly informed as to the recently-appointed nurse having had no training in midwifery or general nursing. We received the following reply from Mr. John Plummer: "I am directed by the Guardians to acknowledge the receipt of your letter dated May 10th, 1895, and to inform you that they are not disposed to answer your queries with reference to the appointment of a nurse, as they do not consider you to be entitled to the same, not being a ratepayer of this union." This we submit to the consideration of the ratepayers of the union, to which, as the writer reminds us, we do not belong. We have little doubt of the matter receiving from the Local Government Board the attention its importance merits.—Ed. *T. H.*]

### Indian Nursing Service Applications.

When a nurse applies for a post as nursing sister in the Indian Nursing Service she has to fill up a form stating her age, place of birth, details of training, and various other particulars. She is required to produce certificates of efficiency in nursing from medical officers only. The Indian Nursing Service apparently dispenses altogether with those testimonials from lady superintendents or matrons, to which so much value is attached in other quarters.

The would-be Indian nursing sister has to produce a letter from a lady of position in society, to show that she is in every way a "desirable person to enter a service composed of ladies of good social position with whom she will associate." Excellent as this precautionary measure may be, if regarded as an additional testimonial, it cannot be held to take the place of one given by the head of the school where the candidate received her training. It is obvious that certificates, possibly given by young medical officers, must need supplementing by the opinion of more experienced persons, if the Indian Nursing Service is to be recruited by the best qualified nurses.

Qualifications.

The regulations now demand that nursing sisters must have had three years' training and service combined, in a general hospital where there are sisters, and where male patients receive medical and surgical treatment.

The nursing sisters are engaged for a term of five years, renewable for a further similar term if desired by Government and by the lady nurse. Six months' notice on either side can, however, terminate the engagement, subject, of course, to certain restrictions. The rules as to pay and leave are accurately defined under the heading of "Conditions of Appointment" on the forms provided for candidates, and anyone entering the Indian Nursing Service is able to form a fair estimate of the prospects that await her.

### Where to Go.

St. Marylebone Parish Church.—Handel's "Messiah" will be performed on May 30th at eight p.m.

St. George's Hospital Museum.—The annual exhibition of the Graphic Society (which is limited to the work of members) will be opened on May 28th at half-past two. On this and following days visitors will be admitted on presenting their cards.

### Notes and Queries.

The contents of the Editor's Letter-box have now reached such unwieldy proportions that it has become necessary to establish a hard and fast rule regarding Answers to Correspondents. In future, all questions requiring replies will continue to be answered in this column without any fee. If an answer is required by letter, a fee of half-a-crown must be enclosed with the note containing the enquiry. We are always pleased to help our numerous correspondents to the fullest extent, and we can trust them to sympathise in the overwhelming amount of writing which makes the new rules a necessity. Every communication must be accompanied by the writer's name and address, otherwise it will receive no attention.

### Queries.

(144) *District Nurse.*—How long training is required for this work?—*Nurse Clara.*

(145) *Stewardess.*—Can you tell me how I can get a post as stewardess on board a ship?—*Constant Reader.*

(146) *Training.*—I should be glad to know if a nurse who paid for her training in a provincial hospital would hold as good a financial position as one trained in a London hospital?—*Edith.*

### Answers.

(144) *District Nurse (Nurse Clara).*—At least two years' training in a good general hospital is desirable, and should be followed by six or twelve months' training in district work.

(145) *Stewardess (Constant Reader).*—Apply to the shipping offices, of which you will find the addresses in the daily papers, for form of application. We presume that you have seen the paragraphs which have appeared from time to time in our columns since The Hospital first advocated the employment of trained nurses in the capacity of stewardesses.

(146) *Training (Edith).*—What do you mean by "financial position?" The expression needs explanation. Assuredly a nurse trained at a large provincial hospital is eligible for future appointments if she has earned a good certificate. The fact of her having paid for her training is a secondary matter. The size of the hospital and the kind of reputation it has as a training school are the chief matters to consider. "Burdett's Hospital Annual" gives particulars of hospitals and county infirmaries, which you would find helpful.

### The Book World for Women and Nurses.

[We invite Correspondence, Criticism, Enquiries, and Notes on Books likely to interest Women and Nurses. Address, Editor, The Hospital (Nurses' Book World), 428, Strand, W.C.]

A June Romance. By Norman Gale. (Rugby: George C. Over. 1894.)

Mr. Gale has shown a certain wisdom in choosing an attractive title for an unattractive work. The diary it is, and nothing else, of a sentimental, mawkish young man, only for "young man" read schoolboy. In these interesting records of his daily life we learn the hero is acting in the capacity of a private tutor at a big country house, "the Romance" centering round the pupil's sister. Interspersed among the pages are several verses, wherein the writer shows an ease and a poetic rhythym. The second of these, written in honour of "unseen Alice Ellaby," is dedicated to the heroine of the book, to whom we are later introduced. Of her first appearance the infatuated lover writes: "While I was watching a dewdrop slide from the ridge of a petal down into the heart of a white rose I suddenly saw the girl." Her slippers he describes as "shining"; her hair a dead-leaf hue, "yet with a glorious glaze of young years upon it, such a polish as the laurel wears." This month of June on which "the Romance" is based is made up of descriptions of how the tutor spent his time. The diary records amongst other things the planning of skeletons for novels, milking cows, and an illness, taken in the most serious of manners, for which strong cups of tea from feminine hands, ice "telegraphed for," make recovery a certainty. We forgot the "literary" orders for society verse from London editors to be "a judicious cross between Wordsworth and Ashby Sterry!" These items sum up the records of the tutor's existence as he records it, The scheme works up to the moment when the lady with the glazed hair receives with open arms the declaration made her by the poet-invalid-tutor, and then the volume abruptly concludes.

### MAGAZINES OF THE MONTH.

As usual in the case of the Westminster Review, the articles are full of a substantial and educative interest. Unlike one or two of its contemporaries among the more serious periodicals, it maintains its character of dealing with the serious questions of the day in a serious manner. Amongst such subjects in the present number are "Some Modern Ideas About Marriage" and "International Agreements and the Sufferers in War," whilst Mr. Todhunter gives us a long review on the "Foundations of Belief." Another article, which claims our attention as being written in a spirit of impartiality and mercy, is by Mr. Rayleigh Vicar on the question of "Capital Punishment"; a punishment with which he is not in sympathy, though he carefully weighs the arguments in its defence. In a lighter strain is a charming description of the little out-of-the-way market town of Ampthill in Bedfordshire; its history, past and present, being picturesquely described by C. Wynn Williams.

The particular article which attracts our attention in the Humanitarian for May is "The Problem Play," as discussed by Robert Buchanan, Sydney Grundy, Dorothy Leighton, Mr. G. Bernard Shaw, and others. The views of the latter are characteristically defined: "What I am prepared to do is to say what I can with the object of bringing some sort of order into the intellectual confusion which has expressed itself in the conundrum." The conundrum being the question here discussed, whether social problems should be freely dealt with in the drama. Among other articles of interest are Mr. George Ives' "A Socialist View of Liberty," "Proportional Local Option," and "Evolution and Heredity," by Dr. Symes Thompson.

In this month's issue, Cassell's Family Magazine contains an account of "Notable Keys" profusely illustrated, amongst which representations are the keys of the Prime Minister's despatch box, Richelieu's key, Mary Queen of Scots', and those of the Bastille. And charmingly are these depicted to us; but it is indeed true, as the writer says, that keys in our own days have not only lost their former beauty, but are fast losing their significance as well. We note that there is no falling off in the improved appearance and contents of Cassell's Magazine, the illustrations for the May number being especially good.

We have just spoken of the improvement in the illustrations of Cassell's Family Magazine; the same comments may be made with equal truth of the Leisure Hour, those in the May issue being extremely good. There is an article on "Childerland Sketches," by Elsa D'esteree Keeling, which alone would make an attractive number, so gracefully and charmingly is it written.

### For Reading to the Sick.

### THE POWER OF SUFFERING.

**Motto.**

Neither our own power nor the world's help can we know without trial. —*Thomas Lynch.*

**Verses.**

Ye noble few! who here unbending stand
Beneath life's pressure, yet bear up a while,
And what your bounded view, which only saw
A little part, deem'd evil, is no more.
The storms of wintry time will quickly pass,
And one unbounded spring encircle all. —*Thomson.*

I know Thee who hast kept my path, and made
Light for me in the darkness, tempering sorrow,
So that it reached me like a solemn joy.
—*Browning.*

I shall know by the gleam and glitter
Of the golden chain you wear,
By your heart's calm strength in loving,
Of the fire they have had to bear.

Beat on, true heart, for ever!
Shine bright, strong golden chain;
And bless the cleansing fire
And the furnace of living pain. —*A. Procter.*

Grief should be the instructor of the wise:
Sorrow is knowledge; they who know the most
Must mourn the deepest. —*Byron.*

By Thy command where'er I stray,
Sorrow attends me all the way,
A never-failing friend:
And, if my sufferings may augment
Thy praise, behold me well content—
Let sorrow still attend. —*Cowper.*

**Reading.**

I am quite sure that sufferers have a wonderful appointment and ordination. No men give me the notion of the privilege of being priests unto God as they; and kings, too, they are, in a sense, though a less obvious one. You do not know the pains and temptations of doing, the self-accusations of strong health, the sense of weakness of the spirit when the flesh is strong. These are struggles within me, and they seem to touch the will more nearly than the others, though I feel assured all the same that, were I called to your task, I should perform it most miserably; at least I should need a degree of grace which has not yet been vouchsafed to me. As for your thought that the sufferer lives less for others than the doer is a mistake, though a very natural one. We shall know more about it hereafter, but I suspect the Cross will be found to have been the great power of the world, both in the members and the Head. —*F. D. Maurice.*

Although this present life be burdensome to our feelings, it is now by Thy grace made very gainful.—*Thos. à Kempis.*

### The Royal British Nurses' Association.

[We have reason to believe the following necessarily imperfect report fairly represents the proceedings at two recent meetings of this association from which reporters were excluded.]

### GENERAL COUNCIL.

At the quarterly meeting of the General Council held on April 19th, the Chairman, Sir James Crichton Browne, read the following letter from her Royal Highness Princess Christian :—

Ladies and Gentlemen,—Never since the honour was conferred on me of being President of the Royal British Nurses' Association have I been absent from the meetings of the Executive Committee and of the General Council, save when prevented by unforeseen and unavoidable circumstances. I had looked forward to attending the Council which meets to-day as a pleasure to myself and as the fulfilment as well of my duty as President. I am, however, debarred from doing so by the absolute disregard of the proprieties of debate, and the introduction of personal attacks upon officers of the Association which have characterised the recent meetings of the Executive Committee, and I must, therefore, make my views known by this written communication. In the first place, I have to say that since the last meeting of the General Council I have received a letter from the Nurse Honorary Secretary, in which that lady has conveyed to me her decision to resign her office. I, on my part, having most carefully taken into consideration all the attendant circumstances, signify to you my assent to that step. Your chairman will, in my name, nominate the lady whom I think most suited to be her successor, and whom I trust the Council will elect. Then I wish also to make it known that it is foreign to my intention to occupy the position of President of the Corporation without taking, as I have always done heretofore, an active part in its discussions and administrative proceedings, and that I therefore express my hope that in future I shall encounter no obstacles to devoting my personal assistance and my best energies to the great work which I have always had so much at heart.

Helena, President.

The Chairman expressed the wish of the Council to lay to heart the lesson contained in this message from their President.

Dr. Bedford Fenwick remarked on the absence from the agenda of certain resolutions which had been sent in to the secretary, and threw doubts on the correctness of her statements in the matter.

The balance-sheet for the year ending March 31st was submitted by the Treasurer, and also a report in which he explained that expenditure and receipts had exceeded their ordinary proportion. In response to special appeals, donations had been given to the Association towards furnishing the new offices, and £626 had been added to the funds by the bazaar. The General Fund had been augmented by something like £150 18s., in response to an appeal for three years' subscriptions. The increase in expenditure was chiefly due to moving into the new offices, and to the present treasurer having paid all debts up to date.

The adoption of the report was moved by Mr. John Langton, and seconded by Mr. Mark Hovell.

It was suggested by Dr. Bedford Fenwick that the Association would be on the verge of bankruptcy in six months; whilst the Treasurer considered that the funds would last for a year and a half or two years, but he owned that another similar special effort would be necessary at the end of that time, unless the Association were enlarged.

Dr. Bedford Fenwick moved, and Miss Annesley Kenealy seconded a resolution, which was carried, declining to adopt the Treasurer's report, desiring that he should furnish more particulars as to the finances.

The report of the Executive Committee was read, and was considered by Dr. Fenwick as "most incomplete and unsatisfactory."

Mrs. Spencer's resignation of the post of Nurse Hon. Secretary was read, and Miss Stewart, Miss Mollett, and Mrs. Andrews were in favour of her being asked to reconsider her decision. Mrs. Spencer having already twice refused a similar request made by the Executive Committee, the Chairman explained that it would be out of order for the General Council to ask her reconsideration of the matter.

A letter was read from Princess Christian nominating Mrs. Dacre Craven as Mrs. Spencer's successor.

The meeting was adjourned until May 10th.

### THE ADJOURNED MEETING.

At this meeting held May 10th, the appointment of Mrs. Dacre Craven was moved by the Chairman, Sir J. Crichton Browne, on behalf of Princess Christian (who was present on this occasion) and seconded by Dr. Priestley, the resignation of Mrs. Spencer having first been accepted by the Council. The Treasurer furnished the report which had been demanded at the last meeting. This showed the increased expenditure was chiefly due to the higher rent, rates, and taxes of the new offices. These, however, it was remarked, were not more expensive than those which Dr. Fenwick had wished to secure for them in Harley Street. The Treasurer considered Dr. Fenwick's prophecy that the Association would be on the verge of bankruptcy in six months was calculated to bring about the very thing he professed to dread. He also showed the excess of expenditure over income was after all comparatively less last year than in the preceding ones if the change of premises were allowed for.

The treasurer's report was afterwards adopted unanimously.

The Chairman called upon Mrs. Bedford Fenwick for the notice which had appeared on the agenda in her name. In her absence from the meeting Dr. Fenwick read a statement to the effect that it had been intimated by Princess Christian to some nurse members of the Executive Committee that her Royal Highness desired to know the evidence on which they had prepared to bring before the Council a statement concerning the business of the Corporation, that she might be enabled to form an opinion upon it. The command of the Princess would be obeyed without delay, and, until this was done they were, Dr. Fenwick said, "of course precluded by the deep respect which they have ever felt and expressed for her Royal Highness from bringing their statement before the Council."

Dr. Fenwick next moved a resolution with regard to the subject of the editorship of the *Nurses' Journal*, which found no seconder.

A second resolution concerning a requisition for a general meeting was also brought forward by Dr. Fenwick, and after an explanation from the Chairman it was asked by Mr. John Langton if any correspondence had taken place on these subjects. Some letters were thereupon read by the Honorary Medical Secretary which showed that legal proceedings had been threatened if the secretary failed to place certain notices upon the agenda. Audible expressions of disapproval accompanied the hearing of the letters, and the Honorary Medical Secretary explained that the secretary was not personally responsible for the placing of any notices on the agenda.

Dr. Fenwick expressed his regret if the tone of the letters, which were written in heat, was offensive.

A resolution was proposed by the Honorary Medical Secretary and seconded by Mr. John Langton, "That this Council places implicit confidence in its secretary." This being put to the meeting was carried enthusiastically. It was stated by the Chairman on behalf of Princess Christian that it was her Royal Highness's intention to take as active a part in the affairs of the Association as she had done hitherto.

The next quarterly meeting of the Association will take place on July 12th at five p.m.

## Figures and Tables

**Fig. 29 Fig. 28 f1:**
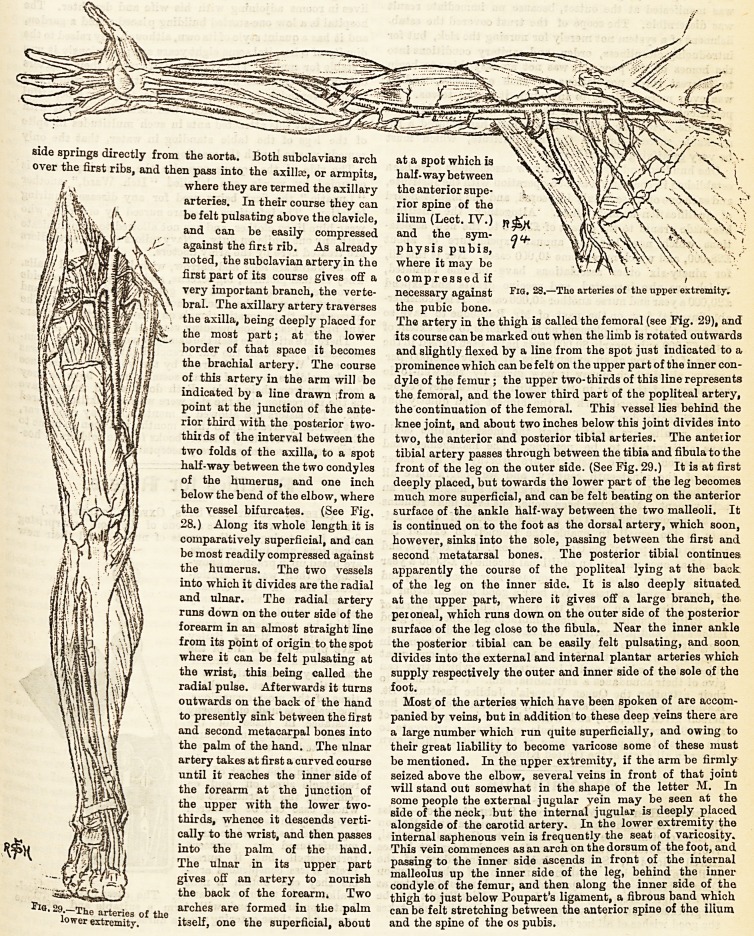


**Figure f2:**